# An alternative reverse genetics system for PRRS virus and its application to define the role of endocytic sorting signal in GP3 protein intracellular trafficking

**DOI:** 10.3389/fmicb.2026.1791468

**Published:** 2026-03-10

**Authors:** Junyu Tang, Hiep Vu, Dongwan Yoo

**Affiliations:** 1 Department of Pathobiology, College of Veterinary Medicine, University of Illinois at Urbana-Champaign, Urbana, IL, United States; 2 Department of Animal Science, Nebraska Center for Virology, University of Nebraska-Lincoln, Lincoln, NE, United States

**Keywords:** endocytic sorting, GP3, intracellular trafficking, PRRSV, reverse genetics, tyrosine-based sorting signal

## Abstract

Porcine reproductive and respiratory syndrome virus (PRRSV) glycoprotein 3 (GP3) forms a heterotrimeric complex with GP2 and GP4, which is essential for viral entry and assembly. However, the intracellular trafficking mechanisms governing GP3 localization and incorporation into virions remain incompletely understood. Here, we identified two highly conserved tyrosine-based sorting signals (YxxΦ) within GP3, motifs that mediate adaptor protein–dependent trafficking through the secretory and endocytic pathways. To define the functional roles of these motifs, we established a Linear Overlapping Infectious Polymerase Amplicon (LOIPA)–based reverse-genetics system for PRRSV. This system enabled precise reconstitution of full-length viral genomes from overlapping cDNA fragments and facilitated rapid introduction of site-specific mutations without bacterial cloning. Using LOIPA, we generated a set of recombinant PRRSV mutants carrying targeted substitutions within the two GP3 YxxΦ motifs. Mutation of Y108A in the YAWL motif at positions 108–111 disrupted GP3 sorting to downstream ER–Golgi intermediate compartments (ERGIC) and markedly reduced infectious virion production. In contrast, mutations in the YVDI motif did not alter GP3 trafficking patterns but exerted limited effects on viral replication, suggesting an indirect regulatory role. Interestingly, the ectopic monomeric expression of GP3-Y108A showed similar trafficking patterns to those of GP3-WT. These results provide novel insights into the molecular interplay between PRRSV envelope proteins and host trafficking machinery, contributing to a deeper understanding of PRRSV assembly, virion morphogenesis, and secretory dynamics. Our study also established LOIPA as a rapid and bacteria-free reverse genetics system for PRRSV, which is readily applicable to other member viruses in the family *Arteriviridae*, enabling functional interrogation of viral genes and rational engineering to produce mutant viruses.

## 1 Introduction

Porcine reproductive and respiratory syndrome virus (both PRRSV-1 and PRRSV-2 genotypes, collectively PRRSV) is an enveloped, positive-sense RNA virus belonging to the family *Arteriviridae*, subfamily *Variarterivirinae*, genu*s Betaarterivirus* (Snijder et al., 2013; Brinton et al., 2021). The PRRSV virion is composed of eight structural proteins that together coordinate genome encapsidation, envelope formation, and virion morphogenesis. The nucleocapsid protein N associates with the viral RNA genome, while the viral envelope incorporates the glycoproteins GP2, GP3, GP4, and GP5, along with the membrane proteins M and E. Based on their relative abundance in mature virions, N, M, and GP5 are considered major structural components, and GP2, GP3, and GP4 are designated minor envelope proteins (de Vries et al., 1992; Veit et al., 2014). In addition to these canonical components, PRRSV encodes a small membrane-anchored ORF5a protein translated from an alternative open reading frame within the GP5 subgenomic mRNA (Johnson et al., 2011). ORF5a protein is incorporated into virus particles at low abundance and is essential for viral replication (Sun et al., 2013). Among the minor envelop proteins, GP4 and GP2 are viral ligands that bind to the receptor CD163 and are essential for the subsequent uncoating of the virus (Das et al., 2010; Van Breedam et al., 2010; Van Gorp et al., 2010; Welch and Calvert, 2010; Zhang and Yoo, 2015). GP2, GP3, and GP4 are co-translocated in the endoplasmic reticulum (ER), where they undergo N-linked glycosylation and assemble into a disulfide-linked GP2/GP3/GP4 heterotrimer prior to virion incorporation (Das et al., 2010; Veit et al., 2014). Formation of the trimeric complex is required for their coordinated trafficking from the ER to the Golgi apparatus, a prerequisite for productive virion assembly (Wissink et al., 2005). While these studies establish the GP2/GP3/GP4 complex as a functional unit, how this complex is stabilized, retained, and incorporated at assembly sites remains incompletely understood. Among the three minor glycoproteins, GP3 is topologically unique. Unlike GP2 and GP4 which possess classical type I transmembrane domains (Du et al., 2012; Zhang et al., 2018), GP3 contains a short C-terminal hydrophobic region that functions as a weak membrane anchor (Veit et al., 2014; Zhang and Veit, 2018; Lv et al., 2025). Structural and biochemical analyses indicate that this region inserts only shallowly into the ER membrane, allowing GP3 to exist in both membrane-associated and soluble secreted form (Mardassi et al., 1998; Matczuk et al., 2013). This unusual topology suggests that GP3 membrane retention and trafficking are tightly regulated processes. Despite these insights, the determinants that govern GP3 intracellular trafficking and membrane retention remain poorly defined. In particular, the mechanisms that regulate GP3 localization within the ER–Golgi secretory pathway and ensure its availability for incorporation into assembling virions have not been directly characterized. A clearer understanding of how GP3 trafficking is controlled is essential for defining its functional role during PRRSV morphogenesis.

Tyrosine-based sorting signals of the YxxΦ type, where Φ denotes a bulky hydrophobic residue (L/I/M/V/F) and x represents any amino acid, are key determinants of intracellular protein trafficking. These motifs mediate direct interactions with the μ-subunits of adaptor protein (AP) complexes, which control clathrin-dependent endocytosis and post-Golgi sorting between the trans-Golgi network and endosomes as well as lysosomal trafficking (Traub et al., 1996; Nakatsu and Ohno, 2003; Owen et al., 2004; Ohno, 2006). Structural studies have demonstrated that the tyrosine residue serves as the primary determinant for adaptor recognition through specific interactions with the μ-subunit of AP complexes, while the hydrophobic Φ residue stabilizes binding and influences adaptor specificity. Consistent with this hierarchy, mutational analyses show that disruption of the tyrosine residue abolishes adaptor engagement, whereas alteration of the hydrophobic residue typically modulates trafficking efficiency or pathway selection (Marks et al., 1997; Owen and Evans, 1998; Kelly et al., 2008). The YxxΦ motifs have been shown to regulate the localization of multiple viral components, promote their correct incorporation into assembling virions, and contribute to their intracellular trafficking. Such functions have been described for the human immunodeficiency virus (HIV-1) gp41 protein, equine infectious anemia virus (EIAV) gag p9 protein, hepatitis C virus (HCV) core protein, and various coronavirus accessory and envelope proteins, underscoring the evolutionary conservation of YxxΦ-mediated trafficking as a strategy to coordinate viral assembly with host secretory pathways (Puffer et al., 1997; Neveu et al., 2012; Minakshi and Padhan, 2014; Barone et al., 2025; Stephens et al., 2025). Sequence analysis of the PRRSV GP3 protein reveals two conserved YxxΦ-like motifs, suggesting that GP3 may utilize such signals to regulate the intracellular localization of minor envelope glycoproteins. However, the functional relevance of these motifs has not yet been examined. Moreover, PRRSV morphogenesis requires tightly coordinated envelope protein trafficking from the ER to the assembly site, yet the viral determinants underlying this process remain unclear.

Reverse genetics systems, in which the viral genome is manipulated and used to recover infectious progeny, are indispensable tools for the study of RNA virus gene function and for a rational design of attenuated vaccine candidates. The most common reverse genetics for PRRSV is the full-length infectious complementary DNA (cDNA) clone. It relies on cloning the viral genome into several complementary DNA fragments and assembling them into a single clone representing the full-length genome in a bacterial plasmid for propagation in the microbial host. However, the viral cDNA copy as the large foreign sequence is unstable during bacterial amplification. Toxic viral sequences and cryptic transcriptional elements may frequently lead to unintended deletions, sequence rearrangements, or sequence drift during propagation, complicating precise genetic manipulation. To mitigate such instability, bacterium-free assembly strategies have recently been explored. The circular polymerase extension reaction (CPER) and the infectious subgenomic amplicons (ISA) have been developed as alternatives to conventional systems, enabling viral genome assembly through either *in vitro* polymerase-mediated extension or intracellular recombination of overlapping DNA fragments (Mélade et al., 2021; Torii et al., 2021). The latter approach primarily relies on host-cell recombination machinery, and its efficiency and accuracy can vary. Because genome assembly occurs intracellularly, control over junctional precision is limited, and recovery of progeny virus may differ between experiments. Moreover, comparative data evaluating its reproducibility for motif-level mutagenesis in PRRSV remain scarce. To address these technical constraints, we developed a linear overlapping infectious polymerase amplicon (LOIPA) system optimized for PRRSV-2. LOIPA streamlines overlapping-fragment rescue by minimizing auxiliary regulatory elements such as hepatitis delta virus ribozyme (HDR) sequence and incorporating a defined linker to facilitate genome assembly. This simplified architecture reduces unnecessary sequence components and standardizes fragment junctions, thereby supporting consistent genome reconstruction and reliable recovery of progeny virus.

Given the dynamics of membrane association of GP3 and its role within the GP2/GP3/GP4 glycoprotein complex, elucidating YxxΦ-mediated GP3 trafficking and localization is particularly important for understanding PRRSV virion morphogenesis. Using the LOIPA reverse genetics system, we introduced targeted mutations into the YxxΦ motifs and evaluated how the YxxΦ motifs regulate GP3 trafficking and subcellular localization, and productive infection of PRRSV.

## 2 Materials and methods

### 2.1 Cells, viruses, genes, and plasmids

MARC-145 cells were cultivated in Dulbecco’s Modified Eagle Medium (DMEM; Corning Inc., NY) supplemented with 10% heat-inactivated fetal bovine serum (FBS; Gibco, Thermo Fisher Scientific, Waltham, MA, United States). BHK-21 cells were maintained in Minimum Essential Medium (MEM; Corning Inc.) containing 10% heat-inactivated FBS. PAM-Cl3 cells are immortalized porcine alveolar macrophages and were provided by Y. M. Lee (Utah State University, Logan, UT) (Su et al., 2024). PAM-Cl3 cells were maintained in RPMI 1,640 medium (Gibco) supplemented with 10% heat-inactivated FBS, 1 × non-essential amino acids (Gibco), and 250 μg/mL G418 sulfate (Corning Inc.). Cells were incubated at 37°C in a humidified atmosphere with 5% CO_2_.

Two full-length infectious cDNA clones of PRRSV-2 were used in this study. One of the clones pCMV-S-P129 was described previously (Yoo et al., 2004; Lee et al., 2005). It was based on the pCMV vector (Clontech, Takara Bio, Mountain View, CA, United States) in which the internal intron and multiple cloning sites between the TATA box and the viral genome were deleted to minimize non-viral leader sequences. The pCMV-S-P129 construct contains the complete, unmodified PRRSV-2 (P129 strain) genome positioned immediately downstream of the shortened CMV promoter. Another infectious cDNA clone pXJ41-FL13, derived from the North American genotype PRRSV-2 strain NVSL 97-7895 (Han et al., 2017), was also used as the genetic backbone in this study. The predecessor clone FL12 represents the first-generation full-length infectious clone of PRRSV-2, constructed to enable recovery of infectious virus through *in vitro* transcription from a T7 promoter (Truong et al., 2004). FL13 was then developed as a CMV promoter–driven derivative of FL12 to simplify virus rescue and improve plasmid stability (Han et al., 2017).

The GP3 coding sequence was amplified from PRRSV-2 strain P129 and subcloned into the mammalian expression vector pXJ41 by directional cloning using *Eco*RI and *Bam*HI restriction sites. The primers used for GP3 amplification were as follows: forward, 5’-CCGGAATTCGCCACCATGGCTAATAGCTGTACATTCCTC-3’; reverse, 5’-CGCGGATCCCTATCGCCGTGCGGCATT-3’. The forward primer contained an *Eco*RI recognition sequence and a Kozak consensus sequence upstream of the start codon to ensure efficient translation initiation, while the reverse primer contained a *Bam*HI recognition sequence downstream of the coding region. PCR products were digested with *Eco*RI and *Bam*HI and ligated into the pXJ41 vector. The GP3 mutant construct was generated by PCR-based site-directed mutagenesis using mutation-specific primers.

### 2.2 Antibodies

Anti (α)-PRRSV-N rabbit polyclonal antibody (NBP3-12889) was purchased from Novus Biologicals (Centennial, CO). The α-PRRSV-GP3 rabbit polyclonal antibody was generated by immunizing rabbits with a mixture of two immunodominant peptides derived from GP3. These peptides correspond to residues Q^61^AAAEVYEPGRSLWC^75^ and S^83^EDDHDDLGFMVPG^96^C (de Lima et al., 2009). The α-Giantin monoclonal antibody (mouse) (9B6, ab37266) was purchased from Abcam (Cambridge, MA). The α-Calnexin monoclonal antibody (mouse) (GT1563, MA5-31501), the α-LMAN1 monoclonal antibody (mouse) (OTI1E3, MA5-25346), and the α-LAMP1 monoclonal antibody (mouse) (H4A3, MA5-46199) were obtained from Invitrogen (Carlsbad, CA). The α-TGN38 monoclonal antibody (mouse) (2F7.1, NB300-575) was purchased from Novus Biologicals. Alexa Fluor 488–conjugated goat anti-mouse IgG (H + L) secondary antibody (35502) and Alexa Fluor 594–conjugated goat anti-rabbit IgG (H + L) secondary antibody (35560) were purchased from Invitrogen.

### 2.3 Construction of the LOIPA reverse genetics system

Viral genomic sequences were divided into four or five overlapping fragments with approximately 100 nucleotides of overlap to enable homologous recombination. To enhance flexibility and minimize strain-specific cloning requirements, a universal regulatory linker was constructed incorporating the conserved 5’ and 3’ PRRSV-2 terminal sequences flanked by a cytomegalovirus (CMV) promoter and simian virus 40 polyadenylation [SV40 poly(A)] signal. The linker sequence is available in GenBank under accession number PX926988.

Viral genomic fragments and the linear linker were amplified using Phusion High-Fidelity DNA Polymerase (Thermo Scientific, Waltham, MA). Primer sequences used for amplification of genomic fragments and the linker are listed in Table 1. PCR products were purified and treated with *Dpn*I (Thermo Scientific) to remove residual template DNA, followed by purification using the GeneJET PCR Purification Kit (Thermo Scientific) according to the manufacturer’s instructions.

**TABLE 1:** Primers used for LOIPA system construction (5’ > 3’).

P129-F1-F	CGTTACATAACTTACGGTAAATGGCCC
P129-F1-R	CATTCCAATCAAAGGAGGTGTCCAT
P129-F2-F	AATGCTGCTGTTCCCGGAACAAA
P129-F2-R	GCAAGGACTCTGGTCTCAATGACT
P129-F3-F	AGCTTGAGGCTTTTGCTGATACCG
P129-F3-R	GGCCCGGGGGAAAATAAAACTTCAT
P129-F4-F	ATGAGTTCACCGGAAACGGTGAG
P129-F4-R	CACGGAACCATCAAGCACAACT
P129-F5-F	ACACTAAGGGCAGACTCTATCGTTG
P129-F5-R	CCATGATTACGCCAAGCTTGCAT
FL13-F1-F	CGTTACATAACTTACGGTAAATGGCCC
FL13-F1-R	AGAGTGTTTTGGGCGTGTGA
FL13-F2-F	TTCTCCCCTAACACCACTGC
FL13-F2-R	ACCAAGGCTGTAAAAGGCGA
FL13-F3-F	ATGTTTGTGCTATCTTGGCTCACAC
FL13-F3-R	GGTAAGGGGTGAGGACTTGC
FL13-F4-F	CCCTCACCTGTCTGGGAGATT
FL13-F4-R	AGCGAGGAAGCGGAAGAGTC
Linker-F	AATTGGAAGAATGTGTGGTGGATGG
Linker-R	GGTTAAAGGGGTGGAGAGACCGTAAA
pP2-F1-F	ATGACGTATAGGTGTTGGCTCTATG
pP2-F1-R	CATTCCAATCAAAGGAGGTGTCCAT
pP2-F2-F	AATGCTGCTGTTCCCGGAACAAA
pP2-F2-R	GCAAGGACTCTGGTCTCAATGACT
pP2-F3-F	AGCTTGAGGCTTTTGCTGATACCG
pP2-F3-R	GGCCCGGGGGAAAATAAAACTTCAT
pP2-F4-F	ATGAGTTCACCGGAAACGGTGAG
pP2-F4-R	CCGCATGGTTCTCGCCAATTAAAT

BHK-21 cells were seeded in 6-well plates and 16–20 h later, transfected with a total of 2.5 μg/well of purified DNA fragment at equimolar ratios using Lipofectamine 2,000 (Invitrogen, Carlsbad, CA). Intracellular homologous recombination between overlapping DNA fragments would result in assembly of a genome-length cDNA positioned between the CMV promoter and SV40 poly(A) signal, enabling transcription of full-length viral RNA and production of infectious virus.

At 24 h post-transfection, culture supernatants were collected and designated passage 0 (P0). P0 supernatants were used to inoculate porcine alveolar macrophage (PAM-Cl3) cells, and 4-day harvest of culture supernatant was designated passage 1 (P1) virus. Subsequent passages using P1 as an inoculum were prepared in the same way and designated P2 and P3. Viral stocks were aliquoted and stored at -80°C until use.

### 2.4 Viral RNA extraction and RT-PCR/RT-qPCR

Viral RNA was isolated from cell culture supernatant using the GeneJET Viral DNA and RNA Purification Kit (Thermo Scientific) in accordance with the manufacturer’s instructions. The extracted RNA was directly used for RT-PCR analyses. To assess the presence of viral genomic RNA, RT-PCR was performed to amplify the ORF2 region using M-MuLV reverse transcriptase (New England Biolabs) and GoTaq^®^ Green Master Mix (Promega) following the manufacturer’s protocols. The primer sequences used for amplification of the ORF2 fragment were as follows: forward, 5’-GCTTTCACGGAATTTTTGGTGTCC-3’; reverse, 5’-AGCGGAAACCAAAAACAGTACG-3’. In addition, the ORF3 gene was amplified to verify the recovery of GP3 mutant viruses using the following primers: forward, 5’-CTCCATATTTTCCTCTGTTGCAG-3’; reverse, 5’-AGACTCGAACTGAAACATGG-3’. Each reaction mixture contained 12.5 μL of 2 × GoTaq^®^ Green Master Mix, 2 μL of cDNA template, 1 μL each of forward and reverse primers (10 μM), and 8.5 μL of nuclease-free water. Amplification was performed for 30 cycles, with each cycle comprising denaturation at 95°C, followed by annealing at 65°C and extension at 72°C.

For RT-qPCR, the reaction was conducted in a total volume of 25 μL using a QuantStudio™ 3 (Applied Biosystems). Each reaction mixture contained 12.5 μL of SYBR Green Master Mix (Applied Biosystems), 2.5 μL of cDNA template, 1.25 μL each of forward and reverse primers (10 μM), and 7.5 μL of nuclease-free water. Amplification was performed for 40 cycles, with each cycle consisting of denaturation at 95°C followed by annealing and extension at 60°C. The specific oligonucleotide primers for RT-qPCR were synthesized by Eurofins Genomics (Louisville, KY). GP3-specific primers are as follows: forward, 5’-GTTTCGTGGTTTCTCAGGCG-3’; reverse, 5’-GAGCTTTTGCGAATCGTCGG-3’. Viral RNA was quantified using the standard curve.

### 2.5 Site-directed mutagenesis and sequencing

PCR-based site-directed mutagenesis was conducted using the Phusion Site-Directed Mutagenesis Kit (Thermo Scientific) in accordance with the manufacturer’s instructions. All primers used for site-directed mutagenesis are listed in Table 2. To verify the successful generation of the intended mutant viruses, viral genomic RNA was extracted from the culture supernatants of cells infected with the P3 virus. The target region within the GP3 gene was amplified by RT-PCR, and the resulting products were sequenced to confirm the presence of the desired mutations. The PCR products were sequenced at the Roy J. Carver Biotechnology Center, University of Illinois Urbana-Champaign.

**TABLE 2:** Primers used for site-directed mutagenesis of GP3 gene in pP2-F4.

GP3Y108A-F	ACTTGACCAGTGTTGCCGCCTGGTTGGCGTTC
GP3Y108A-R	GCCAACCAGGCG**GC**AACACTGGTCAAGTGGCTT
GP3A109C-F	CAGTGTTTAC**TG**CTGGTTGGCGTTCCTGTC
GP3A109C-R	CGCCAACCAG**CA**GTAAACACTGGTCAAGTG
GP3W110K-F	CAGTGTTTACGCC**AA**GTTGGCGTTCCTGTCCTTC
GP3W110K-R	GAACGCCAAC**TT**GGCGTAAACACTGGTCAAGTG
GP3Y136A-F	GTGAGTGAAGTT**GC**TGTTGACATCAAGCACCA
GP3Y136A-R	GATGTCAACA**GC**AACTTCACTCACGTTCCCTATC
GP3V137C-F	CGTGAGTGAAGTTTAT**TG**TGACATCAAGCACCAATTC
GP3V137C-R	GCTTGATGTCA**CA**ATAAACTTCACTCACGTTCCCTATC
GP3D138C-F	GAAGTTTATGTT**TG**CATCAAGCACCAATTCATCTGCGC
GP3D138C-R	GAATTGGTGCTTGATG**CA**AACATAAACTTCACTCACG
GP3I139G-F	AGTTTATGTTGAC**GG**CAAGCACCAATTCATCTG
GP3I139G-R	GAATTGGTGCTTG**CC**GTCAACATAAACTTCACTC

Bold and underlined nucleotides indicate the substitutions introduced for site-directed mutagenesis.

### 2.6 Virus titration by TCID_50_

MARC-145 cells were seeded into 96-well plates and cultivated to form a uniform confluent monolayer. P3 virus stocks were serially diluted in 10-fold increments, and 0.05 mL of each dilution was inoculated into eight independent wells. After infection, cells were incubated for 4–5 days and monitored for the development of cytopathic effects (CPE). Viral titers were calculated by 50% tissue culture infectious dose. Each sample was evaluated in three independent runs.

### 2.7 Immunofluorescence assay

Virus-infected MARC-145 cells were seeded onto microscope coverslips and cultivated for 24 h. After three washes with PBS, the cells were fixed and permeabilized with 100% methanol for 15 min at 4°C. Subsequently, the cells were blocked with 1% bovine serum albumin (BSA) in PBS for 1 h at room temperature (RT), followed by incubation with primary antibodies diluted 1:200 in PBS containing 1% BSA overnight at 4°C. After three additional washes with PBS, the cells were incubated with secondary antibodies (1:200 dilution) for 1 h at RT. Nuclei were counterstained with DAPI (4’,6-diamidino-2-phenylindole) for 5 min at RT. Finally, the coverslips were mounted onto glass slides using Fluoromount-G mounting medium (Southern Biotech, Birmingham, AL), and fluorescence signals were visualized with a Nikon A1R confocal microscope.

## 3 Results

### 3.1 Development of linear overlapping infectious polymerase amplicon system

The genomes of PRRSV are large in size, structurally complex, and difficult to manipulate. Furthermore, full-length cDNA clones placed into plasmids exhibit frequent instability in bacterial hosts, often resulting in non-viable clones. Conventional reverse genetics approaches typically rely on *in vitro* transcription from plasmid templates to generate full-length viral RNA. However, transcription enzymes are inefficient at copying long templates, which compromises both RNA yield and integrity. Moreover, large RNAs are inherently prone to degradation, further limiting the recovery of intact transcripts. Collectively, these challenges underscore the need for alternative approaches to efficiently rescue infectious RNA viruses.

**FIGURE 1:**
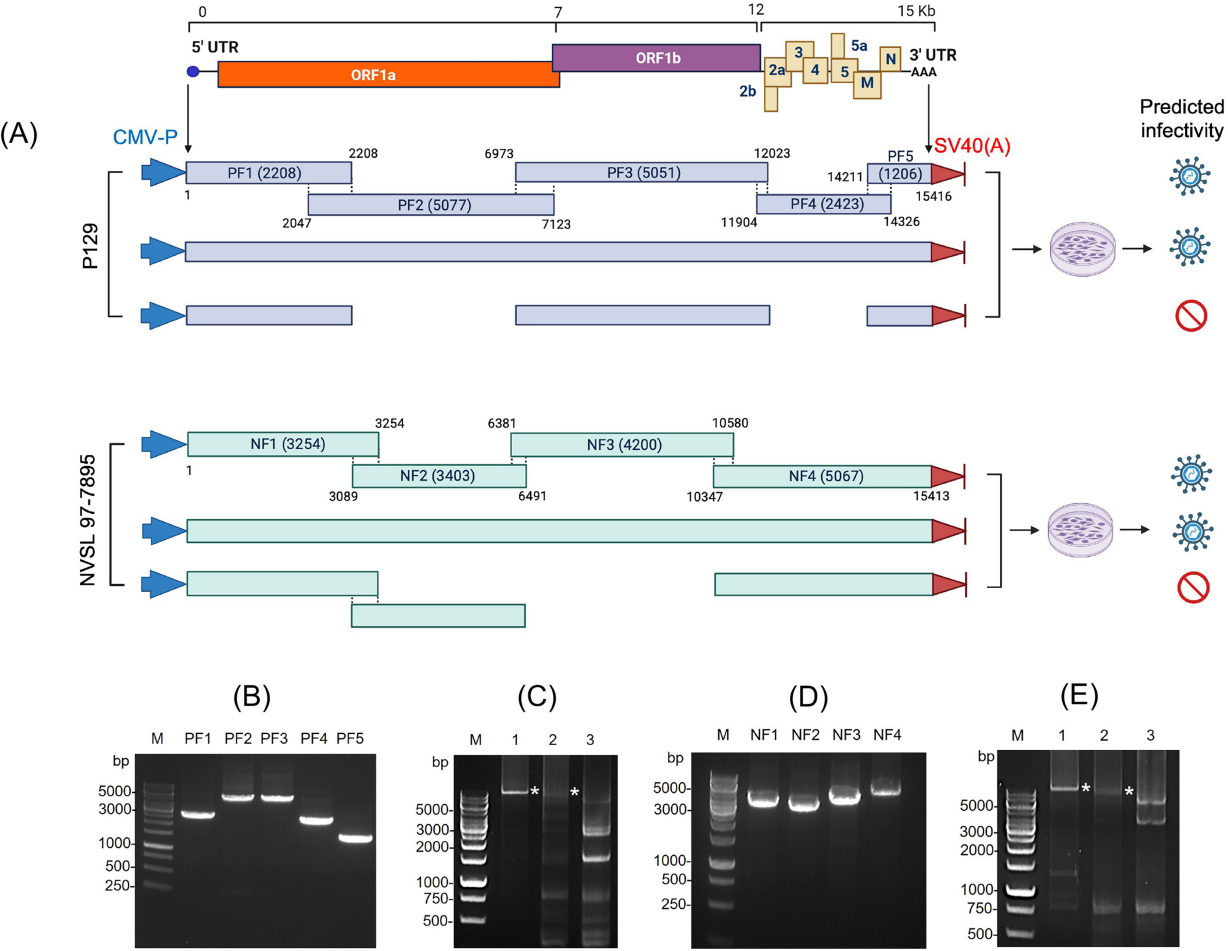
Strategy for the development of the linear overlapping infectious polymerase amplicon (LOIPA) system for PRRSV. **(A)** PRRSV genome organization and schematic workflow for LOIPA development. The viral genome was divided into four (NF1 through NF4) and five (PF1 through PF5) overlapping fragments for two representative PRRSV-2 strains, NVSL 97-7895 and P129, respectively. The 5’-most fragment was placed under the control of a CMV promoter (CMV-P), and the 3’-fragment contained an SV40 polyadenylation signal [SV40 poly(A)]. Predicted infectivity outcomes following co-transfection of fragment combinations are indicated schematically. **(B)** Agarose gel electrophoresis of PCR-amplified fragments (PF1–PF5) corresponding to the P129 genome. PCR amplicons for PF1 (2,732 bp), PF2 (5,077 bp), PF3 (5,051 bp), PF4 (2,423 bp), and PF5 (1,517 bp) are shown. For PF1 and PF5, the gel band sizes include CMV-P and SV40(A), whereas the schematic in **(A)** reflects only the viral genomic regions. **(C)** PCR amplification of P129 LOIPA control groups. Lane 1, the full-length P129 genome clone containing a CMV promoter and an SV40 poly(A) signal; lane 2, the full-length linear PCR product assembled from all five overlapping fragments; lane 3, a partial PCR product assembled from PF1, PF3, and PF5, excluding PF2 and PF4. Asterisks indicate positive controls that yielded successful amplification of the complete viral genome. **(D)** Agarose gel electrophoresis of PCR-amplified fragments (NF1–NF4) corresponding to the NVSL 97-7895 genome. PCR amplicons for NF1 (3,780 bp), NF2 (3,403 bp), NF3 (4,200 bp), and NF4 (5,290 bp) are shown. For NF1 and NF4, the gel band sizes include CMV-P and SV40(A), whereas the schematic in **(A)** reflects only the viral genomic regions. **(E)** PCR amplification of NVSL 97-7895 LOIPA control groups. Lane 1, the full-length NVSL 97-7895 genome clone containing a CMV promoter and an SV40 poly(A) signal; lane 2, the full-length linear PCR product amplified using four overlapping fragments; lane 3, a partial PCR product amplified using NF1, NF2, and NF4, excluding NF3. Asterisks indicate positive controls that yielded PCR products representing the full-length viral genome.

**FIGURE 2:**
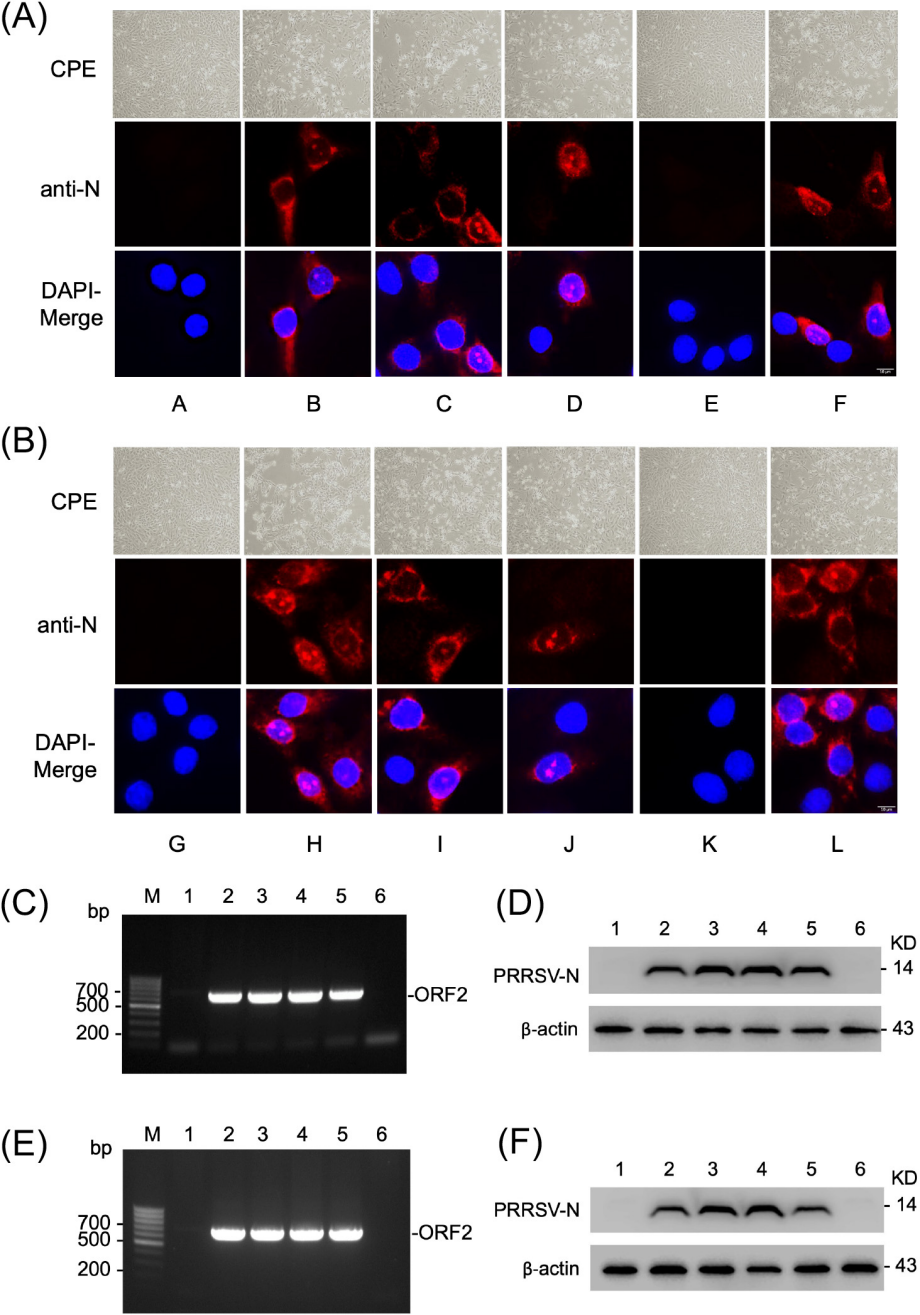
Rescue of infectious PRRSV following LOIPA-mediated genome assembly. **(A)** Rescue of infectious PRRSV strain P129 using the LOIPA system. The upper panels illustrate cytopathic effects observed in P0-inoculated PAM-Cl3 cells. Corresponding immunofluorescence images show expression of the viral nucleocapsid (N) protein (red) at 24 h post-infection, detected with an anti-N antibody. Cell nuclei (blue) were counterstained with DAPI. A, mock control; B, pCMV-S-P129 infectious clone; C, full-length P129 genome construct containing a CMV promoter and an SV40 poly(A) signal; D, genome-length PCR product amplified using five overlapping fragments; E, PCR product amplified using PF1, PF3, and PF5, excluding PF2 and PF4; F, co-transfection of five overlapping PCR fragments. **(B)** Rescue of infectious PRRSV strain NVSL 97-7895 using the LOIPA system. Upper panels show cytopathic effects in PAM-Cl3 cells. Lower panels show immunofluorescence for viral N protein (red) with DAPI nuclear counterstaining (blue). G, mock control; H, pXJ41-FL13 infectious clone; I, full-length NVSL 97-7895 genome construct containing a CMV promoter and an SV40 poly(A) signal; J, genome-length PCR product amplified using four overlapping fragments; K, PCR product amplified using NF1, NF2, and NF4, excluding NF3; L, co-transfection of four overlapping PCR fragments spanning the full-length NVSL 97-7895 genome. **(C)**, RT-PCR detection of PRRSV genomic RNA in PAM-Cl3 cells infected with LOIPA-derived P129 viruses. Viral RNA was extracted from the culture supernatants of P1-infected cells, and the ORF2 gene was amplified by RT-PCR. Bands corresponding to the ORF2 gene (680 bp) are shown. Lane 1, mock control; lane 2, pCMV-S-P129 infectious clone; lane 3, full-length P129 genome construct containing a CMV promoter and an SV40 poly(A) signal; lane 4, genome-length PCR product amplified using five overlapping fragments; lane 5, co-transfection of five overlapping PCR fragments (PF1, PF2, PF3, PF4, and PF5) representing the full-length P129 genome; lane 6, partial PCR product amplified using PF1, PF3, and PF5, lacking PF2 and PF4. **(D)** Western blot analysis of PRRSV N protein in PAM-Cl3 cells infected with LOIPA-derived P129 viruses. Cell lysates were prepared at 24 h post-infection and probed with an anti-N antibody. β-actin served as a loading control. Lanes 1–6 correspond to the experimental groups shown in **(C)**. **(E)** RT-PCR detection of PRRSV genomic RNA in PAM-Cl3 cells infected with LOIPA–derived NVSL 97-7895 viruses. Viral RNA was extracted from the culture supernatants collected from infected cells, and the ORF2 gene was amplified by RT-PCR. Bands corresponding to the ORF2 gene are shown. Lane 1, mock control; lane 2, pXJ41-FL13 infectious clone; lane 3, full-length NVSL 97-7895 genome construct containing a CMV promoter and an SV40 poly(A) signal; lane 4, genome-length PCR product amplified using four overlapping fragments; lane 5, co-transfection of four overlapping PCR fragments (NF1, NF2, NF3, and NF4) representing the full-length NVSL 97-7895 genome; lane 6, partial PCR product amplified using NF1, NF2, and NF4, lacking NF3. **(F)** Western blot analysis of PRRSV N protein expression in PAM-Cl3 cells infected with LOIPA–derived NVSL 97-7895 viruses. Cell lysates were analyzed using an anti-N antibody. β-actin served as a loading control. Lanes 1–6 correspond to the experimental groups shown in **(E)**.

To address these limitations, we established a simple and novel PCR-based reverse genetics system, termed Linear Overlapping Infectious Polymerase Amplicons (LOIPA). For this study, we used two PRRSV-2 strains: P129 and NVSL 97-7895. The genome of P129 was divided into five overlapping fragments (F1 through F5), and that of NVSL 97-7895 into four fragments (F1 through F4), each fragment with ∼100 nucleotide overlaps (Figures 1A,B,D). As positive controls, we included their corresponding infectious clone plasmids pCMV-S-P129 and pXJ41-FL13, respectively. Additional controls included a full-length PCR product generated from all fragments and a partial PCR product lacking one or two fragments, consisting of discontinuous components (Figures 1C,E). Co-transfection of the overlapping genomic fragments resulted in distinct CPE in experimental groups, whereas no CPE was observed in negative controls, confirming successful recovery of infectious virus (Figures 2A,B). Rescue was dependent on inclusion of all required genomic fragments, and incomplete fragment combinations did not produce infectivity. The rescue of PRRSV from the PCR amplicons was validated by independent assays. Immunofluorescence analysis using N-specific antibody showed expression of N protein in P1-infected cells. The N protein was localized in the perinuclear region and subsequently translocated into the nucleus and nucleoli (Figures 2A,B), which represents typical distribution patterns of the N protein in PRRSV-infected cells. RT-PCR analysis of the ORF2 gene using RNA extracted from the culture supernatant confirmed the presence of the viral genome (Figures 2C,E). Furthermore, Western blot analysis demonstrated N protein expression in cells infected with the P1 virus, confirming the generation and infectivity of progeny and subsequent viral propagation (Figures 2D,F). In contrast, no signal was observed for either the ORF2 gene or the N protein in the negative control groups. These data indicate that the LOIPA is a functional reverse genetics system.

The LOIPA system offers several distinct advantages over plasmid-based reverse genetics. First, the use of PCR fragments allows precise control of genome length and sequence fidelity. Second, this system eliminates reliance on bacterial amplification of full-length clones, thereby reducing the risk of unwanted genetic mutations. Finally, the workflow is fast, technically simple and straightforward, and highly reproducible. Together, these features make the LOIPA system a powerful and efficient genetic tool for the recovery and genomic manipulation of PRRSV and potentially other RNA viruses.

### 3.2 Development of a PRRSV-2-specific universal linker for LOIPA system optimization

**FIGURE 3:**
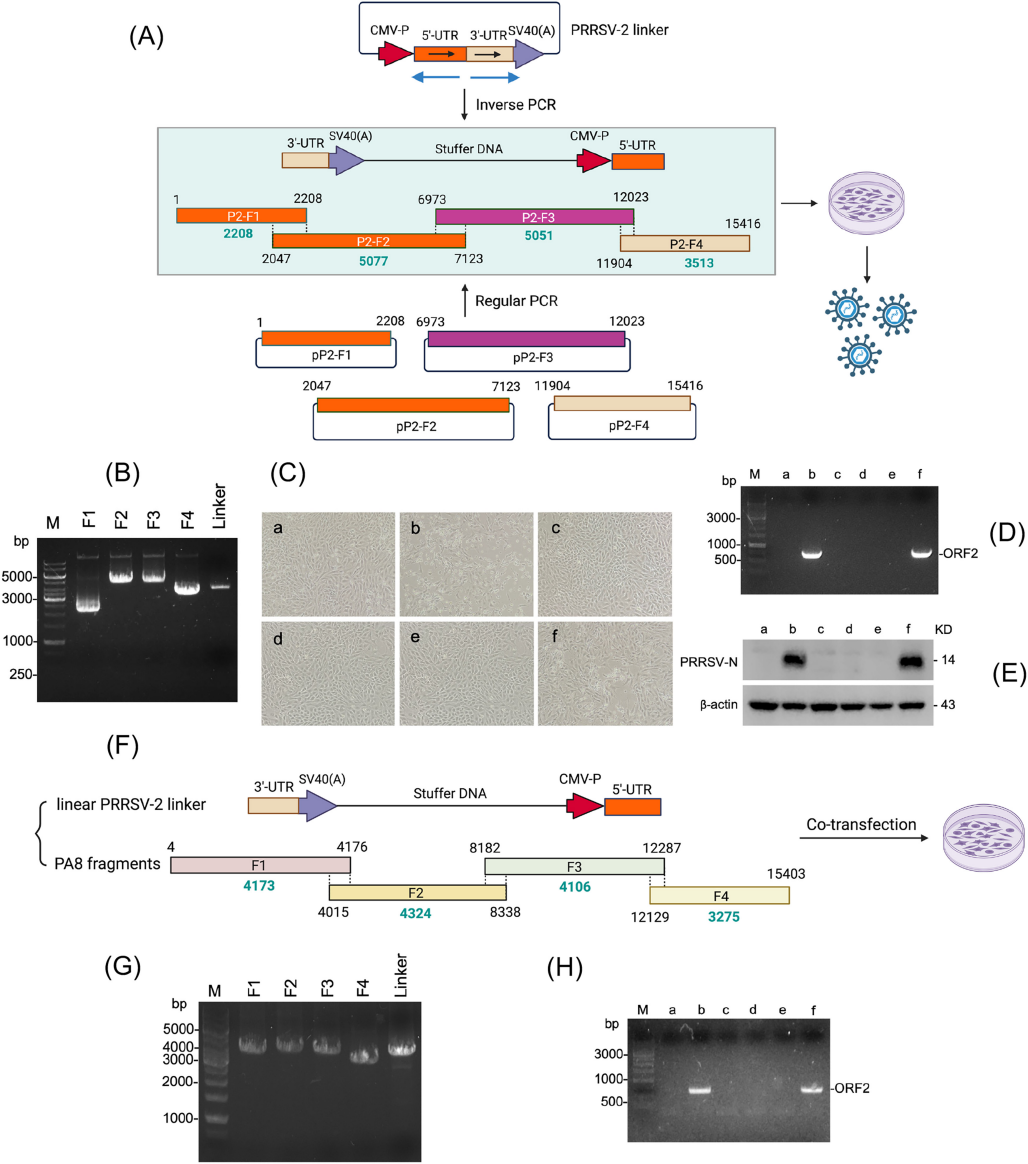
Generation of a versatile PRRSV-2 linker and optimization of the LOIPA system. **(A)** Schematic illustration of the PRRSV-2 linker and optimized LOIPA strategy. Four overlapping genomic fragments (F1–F4) were individually amplified by high-fidelity PCR, and each amplicon was cloned into pCR-Blunt II-TOPO vectors, named pP2-F1 to pP2-F4. The linker was amplified by inverse PCR to generate a linearized template, which was combined with genomic fragments for co-transfection into permissive cells. **(B)** Agarose gel electrophoresis of PCR products amplified from plasmids pP2-F1 to pP2-F4, corresponding to genomic fragments F1–F4, together with the linearized PRRSV-2 linker. **(C)** Cytopathic effects in PAM-Cl3 cells infected with P1 virus generated by the LOIPA system using the universal linker. a, Mock-infected control; b, pCMV-S-P129 infectious clone; c, four overlapping PCR fragments (F1, F2, F3, and F4) representing the full-length genome; d, linker alone; e, linearized linker in combination with three PCR amplicons (F1, F2, and F4), excluding F3; f, linearized linker in combination with all four overlapping PCR amplicons (F1, F2, F3, and F4) representing the full-length genome. **(D)** Detection of the ORF2 gene from progeny viruses rescued using the linker-based LOIPA system. Group labels a-f are identical to those defined in Panel **(C)**. **(E)** Detection of N protein in cells inoculated with progeny viruses rescued using the linker-based LOIPA system. Group labels a-f are identical to those defined in **(C)**. **(F)** Schematic representation of the PA8 genome divided into four overlapping fragments (F1–F4) for assembly using the optimized LOIPA strategy. **(G)** Agarose gel electrophoresis of PCR products corresponding to PA8 genomic fragments F1–F4, together with the linearized PRRSV-2 linker. **(H)** RT-PCR detection of the ORF2 gene in culture supernatants. a, Mock-infected control; b, PA8 infection; c, four overlapping PCR fragments F1, F2, F3, and F4 representing the full-length genome; d, linker alone; e, linearized linker combined with three PCR amplicons (F1, F2, and F4), excluding F3; f, linearized linker combined with all four overlapping PCR amplicons (F1, F2, F3, and F4) representing the full-length genome.

Existing reverse genetics systems often involve laborious cloning steps and strain-specific optimization, which considerably limit their flexibility and throughput. To improve the LOIPA system, we designed a versatile linker element that can be broadly applied across diverse PRRSV-2 isolates. A total of 25 representative PRRSV-2 strains were randomly selected from the GenBank database.^1^ Sequence alignments of their 5’ and 3’ untranslated regions (UTRs) were performed, and highly conserved regions were identified and used for primer design. Using the pCMV vector as a backbone plasmid, the 5’ and 3’ regions were amplified and inserted to generate a 3,806-bp linker construct. The linker integrates the highly conserved terminal sequences of the PRRSV-2 genome flanked by the CMV enhancer/promoter and the SV40 poly(A) signal as transcription regulatory elements (Figure 3A). Upon linearization, the linker functions as a universal regulatory template that can be co-transfected with genomic fragments into permissive cells, resulting in the recovery of infectious PRRSV particles. This modular design facilitates rapid genome assembly, efficient transcription of full-length cDNA clones, and seamless adaptation to different viral strains without the need for strain-specific vector reconstruction. Moreover, the universal linker framework offers a standardized approach for emerging PRRSV variants, enabling comparative functional studies, vaccine vector design, and mutagenesis analyses in a time-efficient manner.

Four overlapping genomic fragments (F1–F4) spanning the P129 genome, together with the universal linker, were amplified by PCR, each yielding a distinct single band of the predicted size (Figure 3B). These fragments were co-transfected with the linearized linker into BHK-21 cells to reconstitute a replication-competent viral genome under the control of the CMV promoter. The rescued virus was subsequently passaged in PAM-Cl3 cells that were permissive for PRRSV. Within 48–72 h post-infection with P1 virus, PRRSV-characteristic cytopathic effects became evident (Figure 3C, panel f), while control groups retained normal cell morphology. This rapid onset of cytopathology highlights the efficiency of recombination and transcription driven by the regulatory cassette of the linker plasmid. RT-PCR targeting the ORF2 gene confirmed *de novo* viral RNA synthesis, with the expected band exclusively in cultures exhibiting cytopathic changes (Figure 3D). N protein was also specifically detected in cell lysates generated using the linearized linker in combination with overlapping fragments (Figure 3E). We also applied the LOIPA system to a field-derived PRRSV-2 isolate, PA8. Viral RNA was extracted and amplified into four overlapping fragments of 4,173 bp, 4,324 bp, 4,106 bp, and 3,275 bp, collectively covering the full-length genome (Figures 3F,G). These fragments were co-transfected with the universal linker into cells using the same protocol. Infectious virus was successfully recovered from culture supernatants, as confirmed by RT-PCR detection of viral RNA (Figure 3H), supporting the broader applicability of the linker-based LOIPA system beyond reliance on pre-established infectious clones. Collectively, these results demonstrate that the linker enables the assembly of the full-length viral genome and autonomous replication of PRRSV-2.

### 3.3 Recovery and phenotypic characterization of GP3 YxxΦ-mutant PRRSV

**FIGURE 4:**
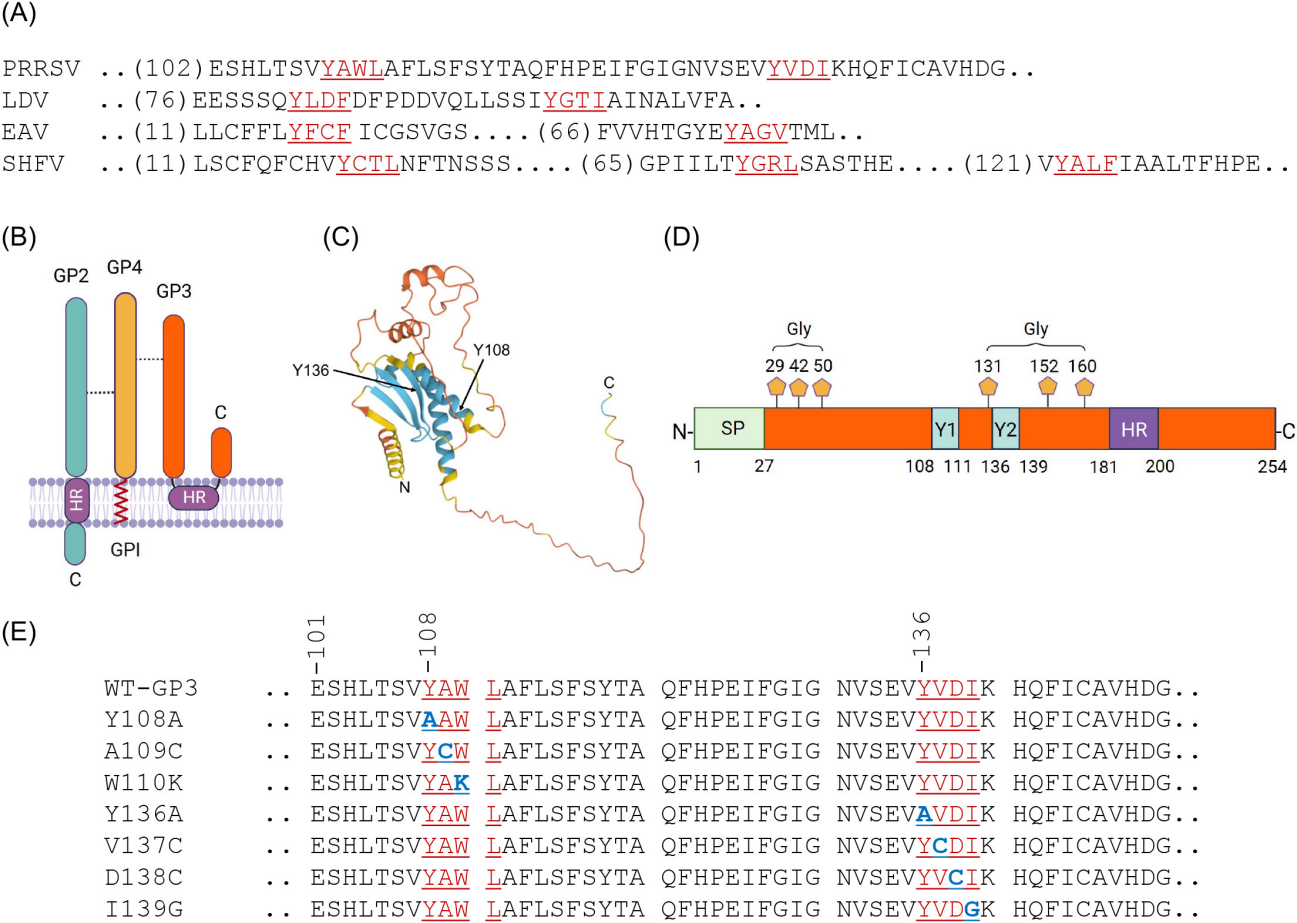
Identification of the tyrosine-based sorting motif (YxxΦ [I/L/M/F/V]) in GP3 of PRRSV-2. **(A)** Sequence alignments of GP3 proteins from four representative arteriviruses, including porcine reproductive and respiratory syndrome virus (PRRSV), lactate dehydrogenase–elevating virus (LDV), equine arteritis virus (EAV), and simian hemorrhagic fever virus (SHFV). Putative tyrosine-based YxxΦ-like motifs are highlighted in red. **(B)** Schematic representation of the PRRSV minor envelope glycoprotein complex. GP3 is associated with GP2 and GP4 on the viral membrane. Predicted transmembrane hydrophobic regions (HR), together with the GPI-anchor signal in GP4 (Du et al., 2012), are illustrated. **(C)** Tertiary structure of the GP3 protein of PRRSV-2 P129 strain predicted by AlphaFold3 (https://alphafoldserver.com/). α-helical and β-sheet structures are indicated in blue. The positions for two tyrosine residues for the motifs are indicated by arrow. **(D)** Structural architecture of PRRSV GP3. The signal peptide (SP), two putative YxxΦ-like motifs (designated Y1 and Y2), an HR, and six predicted N-linked glycosylation sites are shown. Numbers indicate amino acid positions. **(E)** Prediction of the tyrosine-based sorting motif (YxxΦ[I/L/M/F/V]) in PRRSV-2. GP3 proteins of PRRSV were scanned with the Eukaryotic Linear Motif (ELM, http://elm.eu.org/) web server for prediction of the YxxΦ motifs. The conserved YAWL and YVDI core motifs are underlined. To study the roles of these motifs, mutations were introduced to substitute key residues, indicated in blue. Numbers indicate amino acid positions.

GP3 is a minor envelope protein of PRRSV-2. GP3 contains two short sequences resembling the tyrosine-based sorting motifs YxxΦ. The tyrosine motifs are known to function as adaptor-binding signals that regulate transport through the endocytic and secretory pathways. To investigate the role of YxxΦ motifs in PRRSV assembly and morphogenesis, we generated a panel of PRRSV mutants using the LOIPA system and assessed their phenotypic properties during infection. We first conducted bioinformatics analyses of the envelope proteins of arteriviruses using the Eukaryotic Linear Motif (ELM) program.^2^ The GP3 protein of four representative arteriviruses, including lactate dehydrogenase–elevating virus (LDV), equine arteritis virus (EAV), and simian hemorrhagic fever virus (SHFV), appears to contain distinct YxxΦ motifs with species-specific variations in both sequence composition and local topology (Figure 4A). Such interspecies divergence supports the notion that arteriviruses have evolved modular trafficking architectures, balancing conserved sorting principles with host-specific constraints on secretory pathway dynamics. Notably, two canonical YxxΦ motifs were identified in the luminal region of the PRRSV GP3 protein, located at amino acid positions 108–111 and 136–139. These cross-species comparisons were included as a structural reference, and subsequent analyses focused exclusively on PRRSV. We further examined the conservation of these two motifs within PRRSV isolates. Comparative analyses of PRRSV-1 and PRRSV-2 isolates representing each genetic lineage, including six commercial PRRSV-2 modified live vaccine (MLV) strains, showed that the motif at positions 108–111 (_108_YAWL_111_) was fully conserved across all PRRSV-2 isolates. The second motif at positions 136–139 (_136_YVDI_139_) was also conserved but exhibited modest sequence variations (data not shown). These patterns suggest that the motif _108_YAWL_111_ may serve as a primary trafficking determinant, while the motif _136_YVDI_139_ may have auxiliary or strain-specific regulatory functions. The schematic representation of the GP2/GP4/GP3 complex (Figure 4B) illustrates the structural organization of the PRRSV envelope glycoproteins. GP2 and GP4 are canonical type I transmembrane proteins that span the viral envelope. In contrast, the topology of GP3 shows a large luminal domain enriched in N-linked glycosylation sites and a C-terminal hydrophobic region (HR) that anchors the protein to the membrane (Veit et al., 2014). The 3-dimensional structure of GP3, predicted using the AlphaFold3 program,^3^ indicates that Y108 is positioned in an α-helix coiled-coil structure, whereas Y136 positions are in a β-sheet structure (Figure 4C), implicating their importance in structural configuration. The molecular architecture of GP3 delineates the signal peptide (SP), six potential N-linked glycosylation sites, two YxxΦ motifs, and the HR. The two tyrosine motifs are located between the SP and HR within the luminal domain that exposed to the ER and Golgi lumen, where post-translational modification and vesicular sorting occur (Figure 4D). Given their typical positioning and conserved sequence composition, these motifs are predicted to participate in the intracellular transport, folding, or membrane association of GP3, thereby affecting the assembly and secretion of progeny virions. To evaluate the role of these motifs, we generated a series of PRRSV mutants to alter individual residues within the YxxΦ sequences (Figure 4E).

**FIGURE 5:**
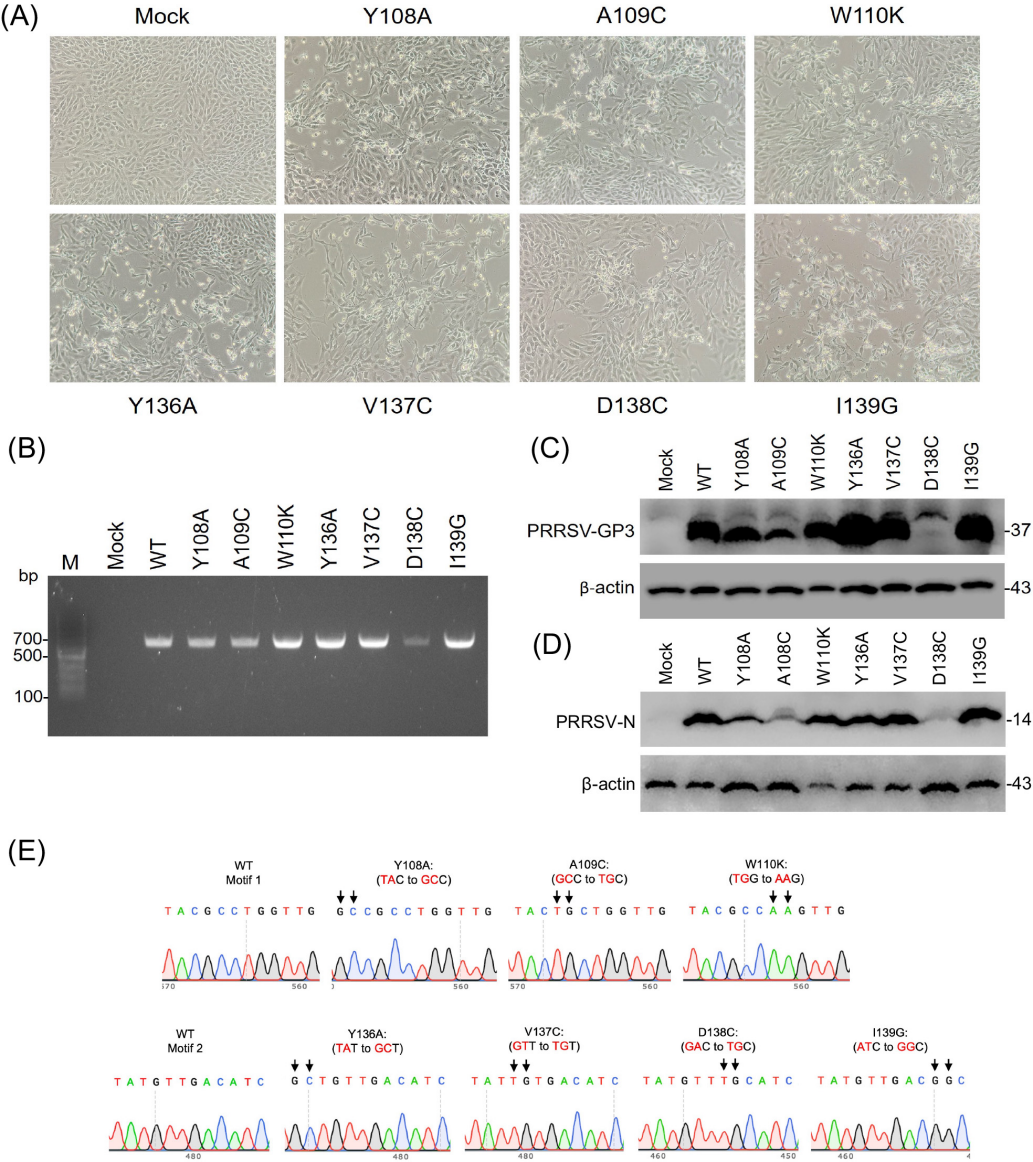
Regeneration of GP3 YxxΦ-mutant viruses. **(A)** Cytopathic effects observed in PAM-Cl3 cells infected with P1 GP3 mutant viruses at 4 days post-infection. Cells infected with individual GP3 mutants exhibited variable degrees of cytopathic effects. Mock-infected cells served as a negative control. **(B)** RT-PCR analysis of the ORF3 gene in rescued viruses. Viral RNA was extracted from the supernatants of P1-infected cells, and the GP3 coding sequence was amplified by RT-PCR. Bands corresponding to the ORF3 amplicon (616 bp) are shown for the WT virus and GP3 mutants. **(C)** Western blot analysis of GP3 protein expression in PAM-Cl3 cells infected with GP3 mutant viruses. Cell lysates were prepared at 24 h post-infection and probed with a polyclonal anti-GP3 antibody. A ∼37 kDa protein band was detected, indicating the generation and infectivity of PRRSV mutants. β-actin was used as a loading control. **(D)** Western blot of viral N protein expression in PAM-Cl3 cells infected with GP3 mutant viruses at 24 h post-infection. The N protein was detected using a specific anti-PRRSV N antibody. β-actin served as a loading control. **(E)** Sanger sequencing confirms the stability of the mutations at two YxxΦ-like motifs of GP3. Chromatograms show the WT sequences and the corresponding nucleotide substitutions for motif 1 (Y108A, A109C, W110K) and motif 2 (Y136A, V137C, D138C, I139G). Mutated positions are indicated by black arrows.

Using the LOIPA system, single amino acid substitutions were introduced to each position to disrupt the motif while minimizing structural disturbance. All mutant viruses were successfully rescued and exhibited cytopathic effects characteristic of PRRSV infection (Figure 5A). The generation of infectious progeny of YxxΦ mutants was confirmed by RT-PCR for the ORF3 gene from corresponding culture supernatants. As shown in Figure 5B, all rescued mutants produced a single amplicon of the expected size, whereas no product was detected in the mock control, indicating successful recovery of PRRSV GP3 mutants. GP3 protein expression was then examined by Western blot. While most mutant viruses expressed GP3 at levels comparable to the wild-type PRRSV, the Y108A and A109C mutants showed a noticeable reduction in GP3 expression, with the most significant decrease observed for the D138C mutant (Figure 5C). To determine whether this reduction was specific to GP3, the viral N expression was examined in parallel. Notably, N protein expression showed similar levels of reduction across the corresponding mutants. These results suggest that some of the YxxΦ substitutions, particularly Y108A, A109C, and D138C, impair overall viral fitness rather than selectively affecting GP3 protein stability (Figure 5D). Sequencing analysis of the GP3 region from P3 virus confirmed that substitutions were retained without reversion or unintended mutations (Figure 5E), demonstrating the genetic stability of the mutagenized genomes. Together, these results confirm that the targeted YxxΦ mutations do not compromise overall genome integrity and validate the LOIPA as a reliable reverse genetics system for studies of PRRSV protein trafficking.

**FIGURE 6:**
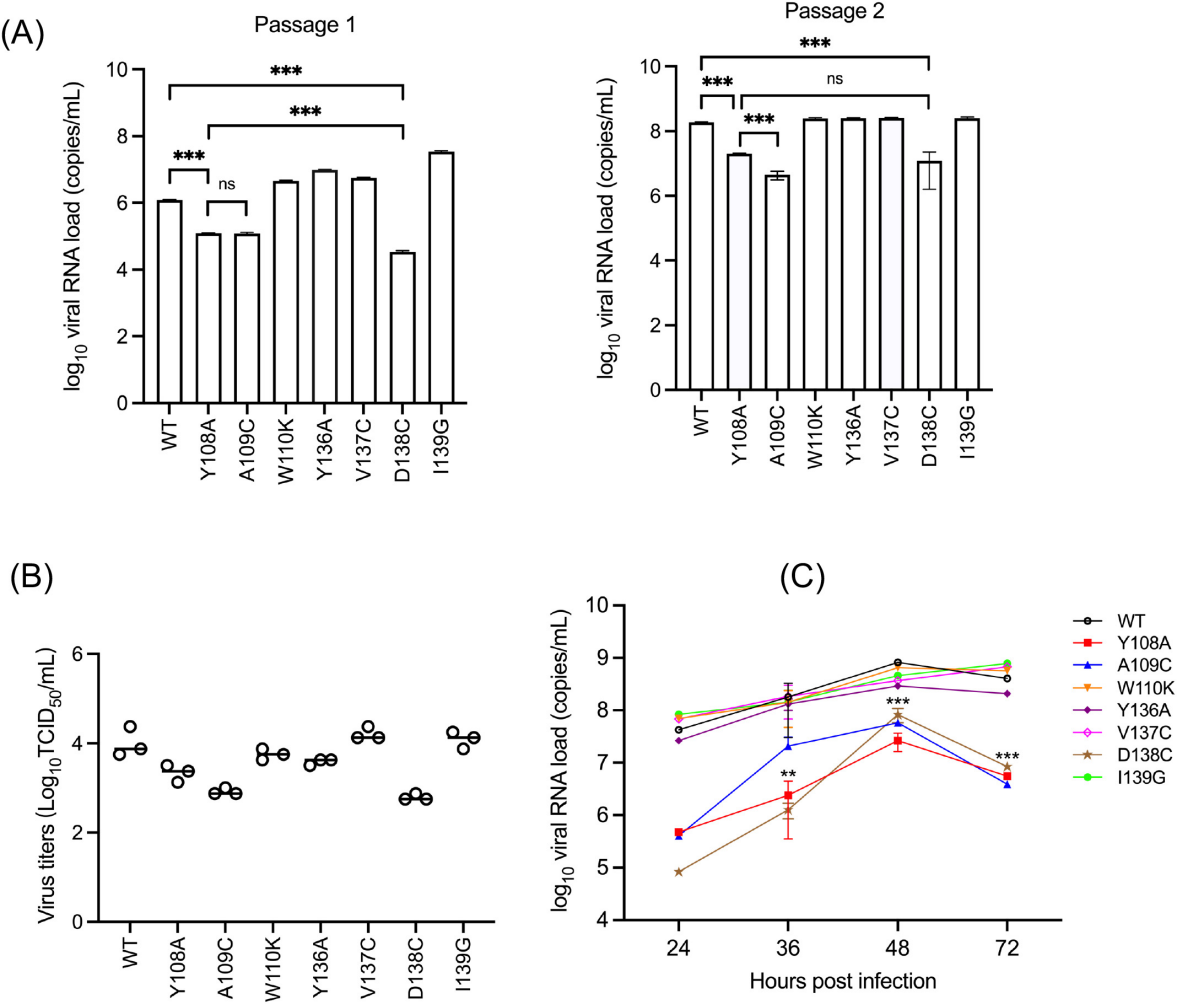
Impact of GP3 YxxΦ mutations on PRRSV replication and infectious virus production. **(A)** Replication of GP3 mutant PRRSVs. Viral RNA level in culture supernatants was determined by quantitative RT-PCR amplifying the GP3 region. A standard curve was generated, and viral copy numbers were calculated as log_10_ copies of cDNA per mL using the standard curve. Data are presented as means ± SEM (standard error of the mean) from three independent experiments. Statistical significance was assessed using Welch’s *t*-test. ***P < 0.001; ns, no significant difference. **(B)** Production of infectious progeny virus was evaluated by TCID_50_ in MARC-145 cells using P3 virus. Viral titers were expressed as log_10_ TCID_50_/mL. Each dot represents an individual biological replicate, and horizontal bars indicate mean values. **(C)** Growth kinetics of GP3 mutant PRRSVs. PAM-Cl3 cells were infected with P2 WT virus or the indicated GP3 mutant viruses. Culture supernatants were harvested at 24, 36, 48, and 72 h post-infection, and extracellular viral genomic RNA was determined by quantitative RT-PCR targeting the GP3 coding region. A standard curve was generated, and viral copy numbers were calculated as log_10_ copies of cDNA per mL using the standard curve. Data are presented as means ± SEM (standard error of the mean) from three independent experiments. Statistical significance between WT and Y108A at individual time points was determined by two-way ANOVA. **P < 0.01; ***P < 0.001.

To examine the significance of the GP3 YxxΦ motif in viral replication, viral RNA in the culture supernatants was quantified by absolute RT-qPCR targeting the GP3 gene. At passage 1 (P1), Y108A mutation resulted in a clear reduction in viral RNA compared with wild-type PRRSV, indicating impaired replication (Figure 6A). A109C showed a similar but less pronounced effect. Among all mutants, the D138C substitution exhibited the most significant reduction in viral RNA, at least during the early passages. Upon further passage, overall RNA levels increased across all groups. At passage 2 (P2), Y108A remained significantly reduced relative to wild type, suggesting a persistent defect associated with the disruption of the tyrosine in the _108_YAWL_111_ motif. Notably, D138C continued to display the lowest RNA levels among all mutants, highlighting its pronounced impact on extracellular viral RNA accumulation. Other substitutions produced modest or negligible effects. To determine whether the differences in RNA loads correlated with the production of infectious virus, virus stocks were titrated by TCID_50_ assay (Figure 6B). The viral titers paralleled the results from RT-qPCR, supporting the consistency between extracellular viral RNA levels and infectious virus yield. These data indicated that disruption of Y108 within the _108_YAWL_111_ motif compromises replication efficiency without abolishing infectivity. Mutations at positions 109 and 138 likewise contribute to optimal viral replication. Accordingly, GP3-mediated protein sorting may modulate replication efficiency rather than serving as a strict requirement for infectious virus production. Mutant virus replication was further assessed by growth kinetics analysis. Viral RNA copy numbers were determined at 24, 36, 48, and 72 h post-infection (hpi) (Figure 6C). Many mutant viruses exhibited similar growth kinetics, with viral RNA levels increasing from 24 to 48 hpi and reaching peak levels at 48 hpi, followed by stabilizing or a modest decline at 72 hpi. Among the mutants, Y108A displayed reduced RNA levels throughout the time course, with significant differences at 36, 48, and 72 hpi, indicating impaired extracellular RNA accumulation.

### 3.4 The canonical Y108-based YxxΦ motif directs GP3 trafficking to ERGIC

To define the role of YxxΦ motif in the intracellular itinerary of GP3, we examined the subcellular distribution of GP3 in cells infected with mutant viruses. Five organelle-specific makers were used to examine the colocalization: LAMP1 representing the late endosome/lysosome, TGN38 for the trans-Golgi network, Giantin for the cis-/medial-Golgi, LMAN1 for the ERGIC, and Calnexin for the ER membrane. The specific antibodies for these organelles allowed us to identify trafficking stages controlled by the YxxΦ motif.

**FIGURE 7:**
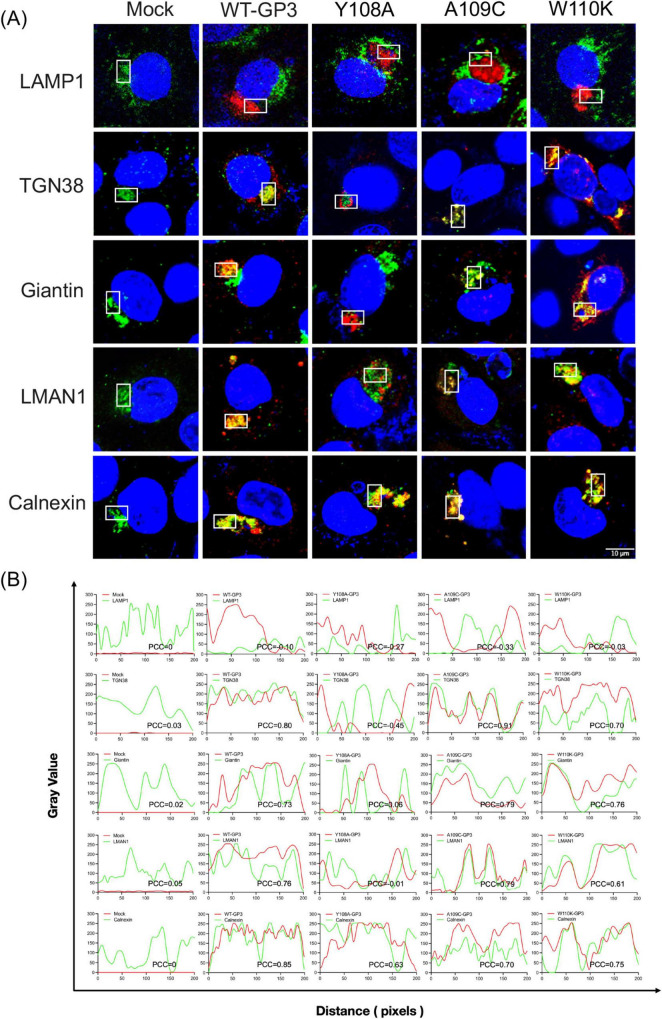
Intracellular trafficking of GP3 in _108_YAWL_111_ mutant PRRSV-infected MARC-145 cells. **(A)** Subcellular localization of GP3 _108_YAWL_111_ mutants. MARC-145 cells were infected with each _108_YAWL_111_ mutant PRRSV at an MOI of 1 for 24 h. Cells were then fixed and stained with a rabbit polyclonal anti-GP3 antibody (red) and mouse monoclonal antibodies against the respective organelle markers (green), as detailed in the Materials and Methods. Nuclei were counterstained with DAPI (blue). Confocal images were acquired using a Nikon A1R microscope. **(B)** Quantitative analysis of colocalization between GP3 _108_YAWL_111_ mutants and compartmental markers. The degree of spatial colocalization (boxes with solid lines in panel A) between GP3 mutants and the respective organelle markers was quantified by line-scan analysis using Fiji Coloc 2. Pixel-wise Pearson correlation coefficients (PCC) were calculated from fluorescence intensity profiles. Values closer to + 1.0 indicate strong positive correlation and spatial colocalization. Values near 0 indicate random or independent distribution, and negative values indicate anticorrelation or mutually exclusive localization patterns. Representative intensity plots and corresponding PCC values for each GP3 mutant are shown.

**FIGURE 8:**
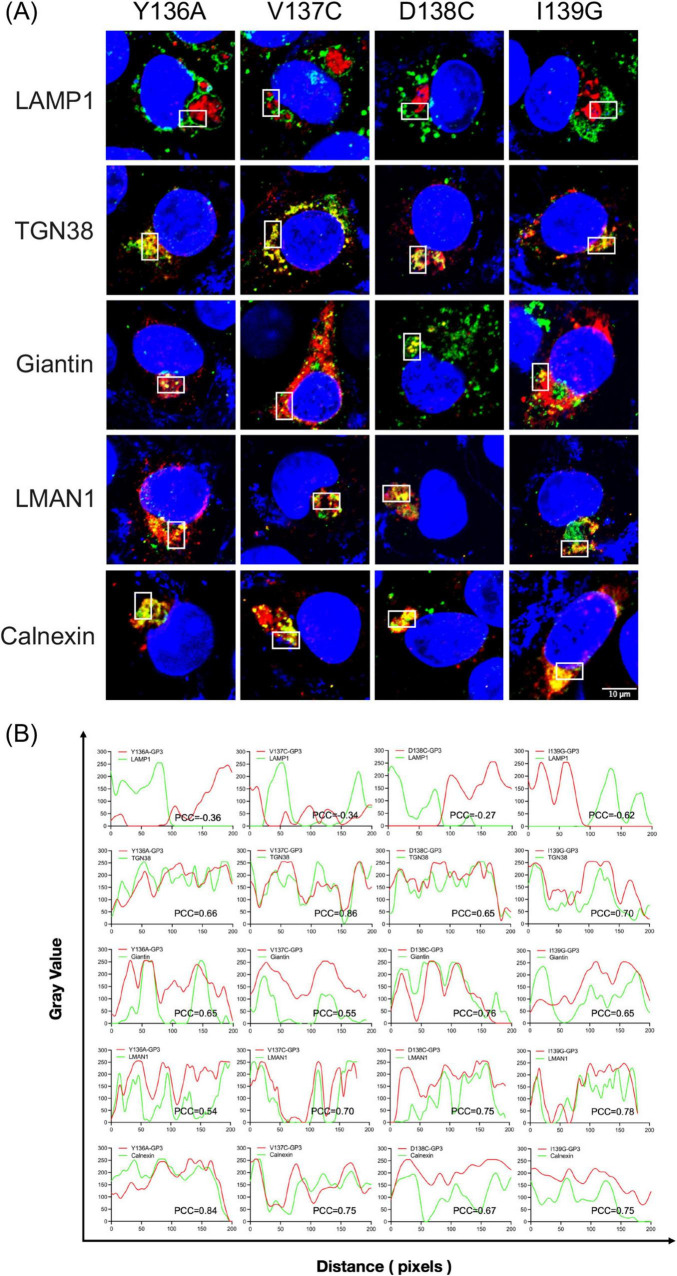
Intracellular trafficking of GP3 in _136_YVDI_139_ mutant PRRSV-infected MARC-145 cells. **(A)** Subcellular localization of GP3 _136_YVDI_139_ mutants. MARC-145 cells were infected with each _136_YVDI_139_ mutant PRRSV at an MOI of 1 for 24 h. Cells were then fixed and immunostained with a rabbit polyclonal anti-GP3 antibody (red) and mouse monoclonal antibodies against the respective organelle markers (green). Nuclei were counterstained with DAPI (blue). **(B)** Quantitative colocalization analysis of GP3 _136_YVDI_139_ mutants with the indicated compartmental markers shown in **(A)** was conducted using the same approach as described above.

In cells infected with wild-type PRRSV, the WT-GP3 protein was strongly distributed to the Golgi and trans-Golgi network, and colocalization with Giantin and TGN38 was pronounced. This is consistent with the well-characterized biosynthetic itinerary of GP3, which travels through the ERGIC into the cis- and medial-Golgi where it associates with GP2 and GP4 to form the heterotrimeric complex essential for virion assembly. Minimal colocalization was observed with LAMP1, indicating limited basal entry into the degradative pathway. Such low-level lysosomal turnover is expected for glycoproteins undergoing transient retrograde–anterograde cycling between the TGN and the endosomal network. These data confirm that GP3 is predominantly committed to the biosynthetic secretory pathway in its native state rather than entry into degradative compartments, as PRRSV envelope proteins are not typically routed through late endosomes. Notably, the Y108A mutant appeared excluded from both downstream degradative compartment and upstream ERGIC and Golgi structures, showing no obvious colocalization with any of the markers tested. In contrast, co-staining with the ER membrane marker Calnexin showed that the Y108A mutant was strongly localized to the ER (Figure 7A). Given that tyrosine-based YxxΦ motifs are recognized by AP complexes at early sorting stations to mediate forward transport toward ERGIC and Golgi compartments, the absence of overlap with LMAN1 is consistent with a failure of Y108A to engage the machinery necessary for export from the very early secretory pathway, leading to retention in the ER or pre-ERGIC intermediates. Therefore, tyrosine at position 108 is required for GP3 to enter the canonical ERGIC–Golgi route. By contrast, the adjacent residues A109C and W110K did not show marked changes in their colocalization patterns compared with WT-GP3. These variants maintained visible overlap with LMAN1, Giantin, and TGN38, with subtle differences in the extent of signal coincidence. Likewise, only a negligible proportion of GP3 entered LAMP1-positive compartments, supporting that endolysosomal routing is not a significant trafficking pathway. Substitutions within _136_YVDI_139_ did not block Golgi targeting. All four mutants retained substantial colocalization with Giantin and TGN38, suggesting that this region does not function as a critical YxxΦ sorting motif for GP3 trafficking. Direct comparison of Y108A and Y136A further indicates a functional hierarchy between the two tyrosine-containing regions within GP3. Although Y136 occupies the tyrosine position of the putative _136_YVDI_139_ sorting motif, replacing this residue did not reproduce the phenotype observed with Y108A (Figure 8A). Instead, Y136A displayed a distribution closely resembling WT-GP3, with preserved colocalization at the ERGIC, Golgi, and trans-Golgi network. This downstream region containing Y136 may therefore serve as a regulatory element, modulating GP3 stability or residence time without governing ER export or early trafficking.

To further characterize intracellular trafficking of GP3 and its mutants, we quantified colocalization between each variant protein and the compartmental markers using Fiji Coloc 2–based line-scan analysis (Figures 7B, 8B). Fluorescence intensities of GP3 (red) and the corresponding organelle marker (green) were extracted along defined linear regions of interest, and gray-value distributions were plotted against pixel distance to visualize spatial covariance. Pearson correlation coefficients (PCC) derived from pixel intensity profiles were used as an objective metric of spatial overlap (Dunn et al., 2011). WT-GP3 exhibited high PCC values with Calnexin, LMAN1, Giantin, and TGN38, consistent with efficient ER export and orderly progression through the early secretory pathway. In contrast, Y108A displayed markedly reduced PCC values with ERGIC and Golgi markers but elevated association with Calnexin, indicative of defective ER exit and ER retention. Other substitutions maintained substantial or partial overlap with ER–Golgi markers, suggesting preserved trafficking competence to varying degrees. PCC values with LAMP1 were uniformly low or negative across all mutants, indicating negligible routing to lysosomal compartments. Collectively, these quantitative analyses reinforce the imaging data and demonstrate that the two YxxΦ-like motifs differentially regulate GP3 sorting within the secretory pathway.

**FIGURE 9:**
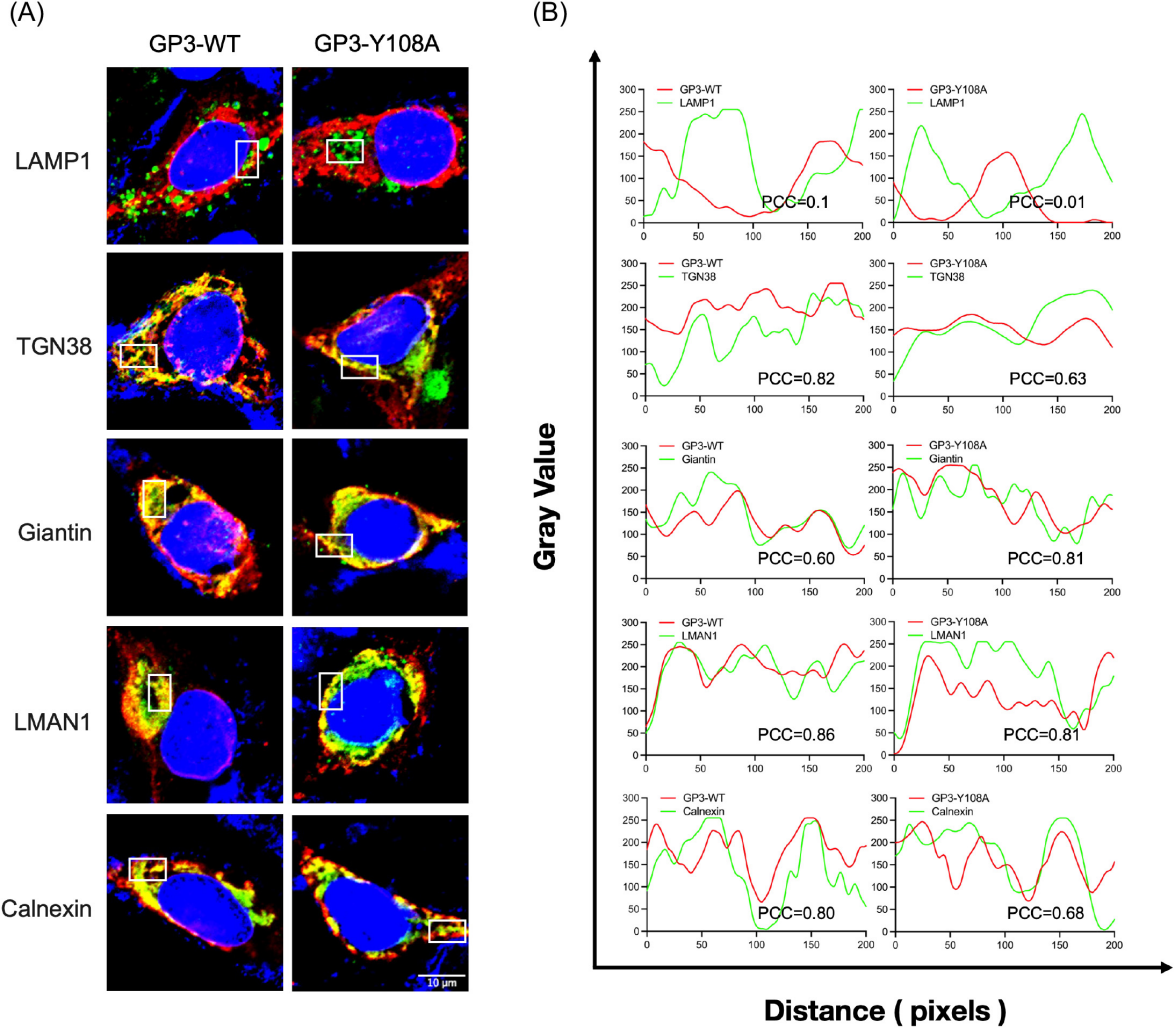
Subcellular localization of ectopically expressed GP3-Y108A in MARC-145 cells. **(A)** MARC-145 cells were transfected with GP3-WT or GP3-Y108A. At 24 h post transfection, cells were fixed and co-stained with rabbit polyclonal anti-GP3 antibody (red) and mouse monoclonal antibodies against the respective organelle markers (green). Nuclei were counterstained with DAPI (blue). **(B)** Quantitative colocalization analysis of ectopically expressed GP3 with the indicated compartmental markers, as shown in **(A)**, was conducted using the same approach as described elsewhere.

Since our analysis was conducted using infectious virus, where GP3 assembles into a trimeric complex with GP2 and GP4, we expressed GP3-Y108A alone by gene transfection and compared its localization with that observed in the heterotrimeric context during infection (Figure 9). When expressed alone as a monomer, GP3-WT traveled efficiently through the secretory pathway and maintained colocalization with ERGIC and Golgi markers. Interestingly, GP3-Y108A did not phenocopy the infection-associated restriction under the controlled setting. Instead, ectopic expression of GP3-Y108A retained appreciable overlap with the secretory pathway. Quantitative analysis supported these observations, as PCC values for GP3-Y108A remained positive with ERGIC and Golgi markers, whereas during infection the same mutant showed reduced correlation with these compartments. These data indicate that the severe trafficking defect of Y108A is apparent during viral infection, highlighting a context-dependent requirement for Y108. While the mutation does not abolish trafficking of GP3 from ER to Golgi when expressed alone, it likely disrupts assembly-competent heterotrimer formation and coordinated export during infection, thereby impairing efficient virion assembly.

**FIGURE 10:**
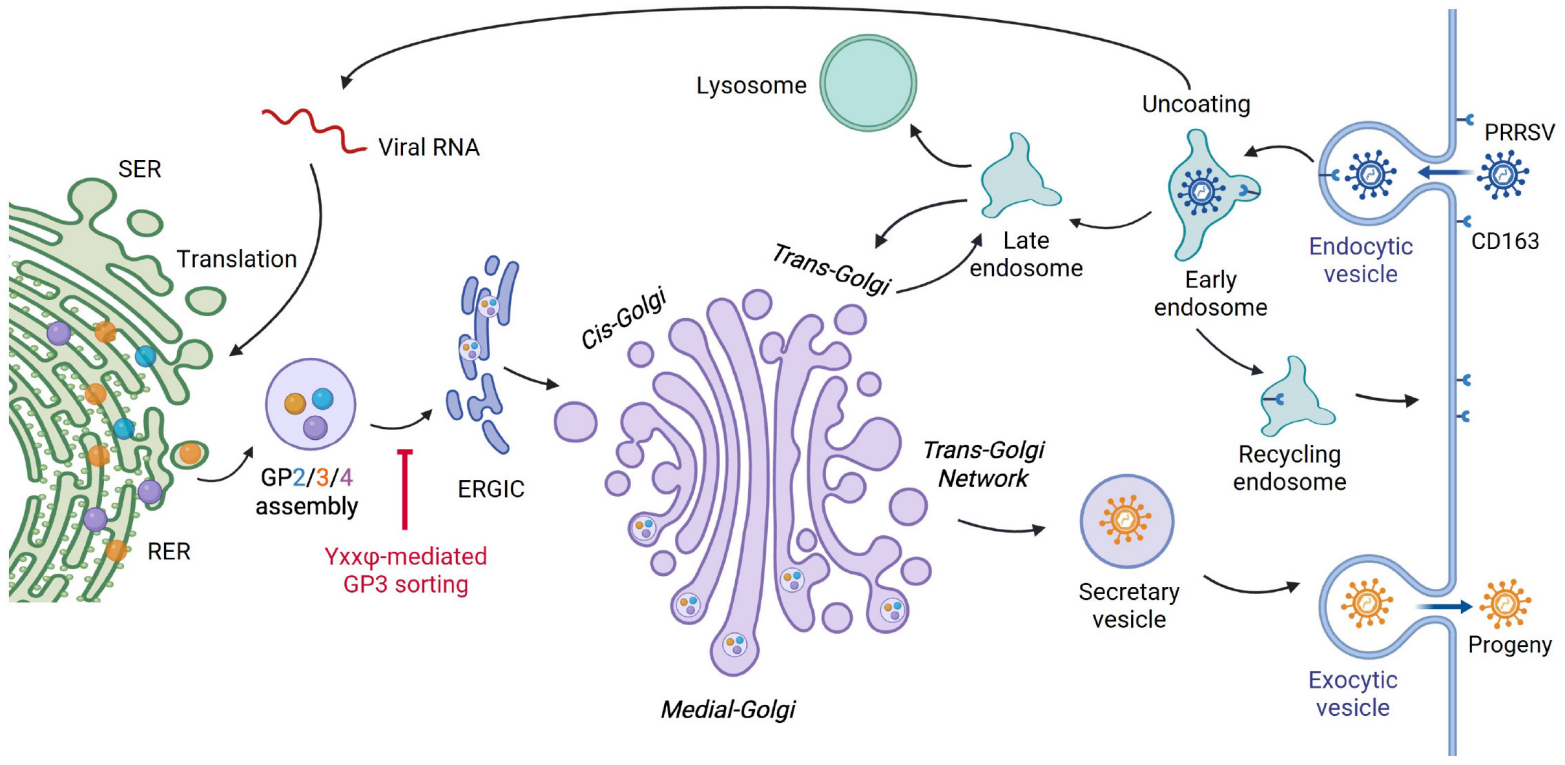
Proposed model of YxxΦ-mediated intracellular trafficking of GP3 during PRRSV infection. PRRSV binds CD163 as the cellular receptor and enters the cell through CD163-mediated endocytosis. PRRSV is transported to early endosomes where uncoating takes place, and CD163 is cycled back to the plasma membrane. Subsequently, the viral genome is transcribed to produce a nested set of 3’-coterminal mRNAs for structural protein synthesis. Membrane proteins are translated in the rough endoplasmic reticulum (RER), where the minor envelope proteins GP2, GP3, and GP4 associate to form a heterotrimeric complex, a prerequisite for their progression to the early secretory pathway. The YxxΦ motif within GP3 functions as a key sorting determinant that governs anterograde trafficking from the ER to the ERGIC and into the Golgi apparatus. Proper recognition of this motif ensures efficient delivery of the GP2/3/4 complex to downstream compartments. As newly assembled virions transit through ER-derived vesicles toward the plasma membrane, a portion of glycoprotein cargo can be routed through retrograde recycling between the Golgi and endosomal compartments, reflecting precise spatial regulation within the secretory system. Collectively, our model implicates the GP3 YxxΦ motif in coordinating early secretory sorting events necessary for efficient glycoprotein trafficking and virion maturation.

Given these compartment-specific phenotypes, we constructed a working model to contextualize the trafficking of GP3 and delineate how the YxxΦ signal manipulates GP3 sorting during the PRRSV life cycle (Figure 10). Following synthesis at the rough ER, GP3 associates with GP2 and GP4 to form the minor glycoprotein complex prior to virion budding. This preassembled complex is subsequently routed through ER-derived membranes and the early secretory pathway, where it converges with the GP5/M heterodimer and the RNA-nucleocapsid protein complex to facilitate virion assembly. The YxxΦ-like motifs within GP3 act as key sorting determinants that promote efficient trafficking of the complex through the ERGIC and Golgi-associated membranes. Perturbation of this sorting signal with the Y108A substitution disrupts normal GP3 routing and compromises the optimal incorporation of minor glycoproteins into assembling virions, resulting in reduced infectivity.

## 4 Discussion

Reverse genetics has become an indispensable tool in molecular virology, elucidating viral genetics, protein functions, pathogenic mechanisms, and evolutionary dynamics. In the case of PRRSV, the first infectious cDNA clone was reported in 1998 (Meulenberg et al., 1998), and since then, multiple reverse-genetics platforms have been developed (Nielsen et al., 2003; Truong et al., 2004; Lee et al., 2005; Fang et al., 2006; Lv et al., 2008; Zhang et al., 2011), allowing precise genome manipulation. These tools have provided a robust experimental foundation for systematic analyses of PRRSV gene function, virus-host interactions, and virulence determinants, and have played a central role in advancing rational vaccine design and evaluation. Despite these advances, critical limitations persist in current PRRSV reverse-genetics platforms. The construction of full-length infectious clones is time-consuming, typically involving sequential subcloning of multiple genomic fragments and extensive *in vitro* ligation procedures. In addition, PRRSV genomes are inherently complex and challenging to manipulate. When cloned into plasmid vectors for reverse-genetics applications, their large genome size and structural features often compromise stability in bacterial hosts. Unwanted adaptive mutations, including point mutations, genomic rearrangements, or spontaneous deletions, may arise during bacterial propagation and thus potentially alter genetic and phenotypic outcomes. Of note, the quasi-species nature, extensive genetic diversity, and high recombination frequency of PRRSV impose additional constraints on conventional reverse-genetics platforms, limiting their capacity to keep pace with the rapid evolution of emerging field strains. In this context, we developed a novel LOIPA reverse-genetics system for PRRSV. LOIPA simplifies full-length infectious genome construction by using long oligonucleotides for assembly, rather than relying on multiple restriction sites and repetitive ligation steps. This system supports precise insertion of reporter genes or targeted mutations with minimal impact on viral replication, while enabling modular exchange of genomic regions to rapidly engineer diverse PRRSV field isolates.

Several bacterium-free strategies have been developed to circumvent the genetic instability associated with bacterial cloning. One early conceptual advance came from the CPER strategy, originally developed for flavivirus and later adapted to coronaviruses. CPER enables the seamless assembly of overlapping viral cDNA fragments together with regulatory elements into a circular full-length genome in a single reaction, followed by direct transfection and intracellular transcription. Multiple studies have demonstrated that CPER supports efficient virus rescue with high sequence fidelity and facilitates rapid introduction of point mutations, deletions, and reporter genes (Edmonds et al., 2013; Piyasena et al., 2017; Tamura et al., 2018; Setoh et al., 2019). Recent methodological refinements have further improved CPER utility. Comparative analyses indicate that CPER avoids many intrinsic drawbacks of BAC- and ligation-based systems while maintaining robust rescue efficiency for ∼30 kb coronavirus genomes (Amarilla et al., 2021). Nevertheless, this technology is not yet widely used for arteriviruses, including PRRSV. Distinct from CPER, which relies on polymerase-mediated *in vitro* genome assembly, the ISA approach reconstructs the viral genome through intracellular recombination of overlapping DNA fragments. PCR-derived fragments spanning the viral genome are introduced into permissive cells, where cellular machinery drives recombination and transcription to generate infectious viruses. ISA-based systems substantially reduce cloning requirements and enable rapid virus rescue within days (Aubry et al., 2014; Atieh et al., 2016; Atieh et al., 2017; Touret et al., 2019; Mélade et al., 2022). A previous study utilized the ISA method to produce recombinant PRRSV (Mélade et al., 2021). In that approach, the human cytomegalovirus promoter (pCMV) was inserted upstream of the first viral genomic fragment to drive RNA polymerase II-mediated DNA transcription. Additionally, the HDR and the SV40 poly(A) signal were subsequently added to the 3’ end of the final fragment to ensure transcription termination and RNA processing. However, this design involved additional regulatory elements and sequential modifications, increasing procedural complexity. Our linker system incorporates only the pCMV promoter and SV40 poly(A) signal, yet it is capable of efficiently promoting viral particle production and replication, even in the absence of HDR. As a result, LOIPA simplifies genome manipulation by incorporating a synthetic linker specifically engineered to drive seamless and directional joining of the terminal fragments. This linker provides defined, high-fidelity complementarity that ensures exact fusion between the 5’ and 3’ ends of the viral genome, yielding a single, continuous, and promoter-bearing template ready for transcription upon entry into cells. Furthermore, it demonstrates broad applicability across diverse PRRSV-2 strains, enabling the rapid and flexible generation of recombinant virus. This streamlined approach offers a robust system for conducting mutagenesis and functional analyses of viral genes and specific motifs, efficiently advancing PRRSV-2 research and development.

During PRRSV assembly, the minor envelope glycoproteins GP2, GP3, and GP4 associate to form a heterotrimeric complex, whereas GP5 forms a heterodimer with the membrane protein M. These two complexes fulfill distinct but complementary roles in PRRSV life cycle, with GP5/M providing the primary structural scaffold that drives membrane curvature and budding, while the GP2/3/4 complex is principally responsible for receptor engagement and viral entry (Wissink et al., 2005). This functional hierarchy implies a degree of modularity during assembly, such that perturbations affecting the trafficking or incorporation of minor glycoproteins may compromise infectivity without necessarily abolishing particle formation. Our findings provide direct experimental support for this model by demonstrating that disruption of tyrosine-based sorting signals within GP3 profoundly alters its intracellular localization yet does not fully prevent viral infectivity. Tyrosine-based sorting motifs of the consensus sequence YxxΦ are typically recognized by AP complexes, thereby directing membrane proteins into vesicles for trafficking between endosomes, TGN, Golgi, ER, or the plasma membrane (Park and Guo, 2014; Tan and Gleeson, 2019). In our study, substitution of the tyrosine residue within the motif _108_YAWL_111_ of PRRSV GP3 resulted in mislocalization of GP3 away from the ER/Golgi region. Quantitative colocalization analyses revealed that the Y108A mutant failed to associate with ERGIC and Golgi markers, indicating that trafficking is arrested before entry into the ERGIC regions. Importantly, two additional GP3 mutants, A109C and D138C, exhibited lower protein abundance than WT and the other variants, yet ER retention was not observed during infection with either mutant. This further supports the conclusion that the pronounced trafficking defect of Y108A cannot be attributed solely to reduced GP3 abundance. Our conclusions regarding PRRSV GP3 are consistent with observations in coronaviruses. In SARS-CoV-2, multiple canonical YxxΦ motifs within the cytoplasmic domain of the accessory protein ORF3a govern its transport through the secretory and endolysosomal pathways. Systematic mutagenesis of these motifs demonstrated that disruption of YxxΦ signals results in pronounced mislocalization of ORF3a away from the cell surface and altered distribution among Golgi, late endosome, and lysosomal compartments (Stephens et al., 2025). Mutation of the YxxΦ motif in the SARS-CoV 3a protein also resulted in Golgi retention, highlighting its role in Golgi-to-plasma membrane trafficking (Minakshi and Padhan, 2014). This similarity suggests that arteriviruses and coronaviruses exploit a shared host-derived trafficking logic to determine envelope protein localization. Nevertheless, the divergent trafficking during infection and ectopic expression indicates that Y108 is not strictly required for the intrinsic export of GP3. When expressed alone as a monomer, GP3 retains sufficient structural integrity and sorting capacity to reach post-ER compartments even with disruption of the YxxΦ motif. Y108A may subtly alter GP3 folding or heterotrimer formation but does not preclude forward trafficking, as transport can proceed independently of viral glycoprotein assembly. During PRRSV infection, GP3 does not function as an isolated cargo but as a component of the GP2/GP3/GP4 heterotrimeric complex. Assembly of this complex occurs within the ER and the early secretory compartments and is coupled to glycoprotein maturation and coordinated targeting to virion assembly sites. In this integrated context, Y108 likely contributes to conformational stability or interaction surfaces required for efficient heterotrimer formation. Impaired heterotrimer assembly would trigger ER retention and delay export, as only properly assembled complexes can efficiently progress through the ERGIC and Golgi and be incorporated into budding virions. Together, these findings support a context-dependent role for Y108 in coordinated glycoprotein assembly rather than baseline intracellular transport. Future studies examining GP2 and GP4 alongside GP3 will further clarify how the Y108A substitution influences trafficking of the entire heterotrimer and whether it ultimately limits viral particle production by reducing the availability of assembly-competent complexes.

Despite the severe trafficking defect during Y108A infection, the virus retained the capacity to produce infectious progeny at reduced levels. Several mechanisms may explain the decreased production of infectious virus. A small fraction of newly synthesized GP3 molecules may still transiently traverse the ER–Golgi interface before misrouting, allowing limited incorporation into assembling virions. In addition, GP3 interacts directly with GP2 and GP4, and these interactions may partially compensate for defective sorting by facilitating indirect recruitment of mislocalized GP3 to assembly sites. As a consequence, progeny virions incorporate reduced levels of GP3 or contain structurally altered GP2/3/4 complexes, yielding particles that are functionally compromised. Importantly, the trafficking defect imposed by Y108A selectively affects envelope maturation, whereas upstream processes such as genome replication, transcription, and nucleocapsid assembly remain intact, explaining why viral replication is attenuated rather than abolished. Phenotypic analysis of GP3 mutants further revealed distinct profiles between the two YxxΦ motifs. Mutation of Y108 within the motif _108_YAWL_111_ caused a moderate but reproducible reduction in replication efficiency, consistent with the role of tyrosine residues as the primary determinants of adaptor engagement. In cellular cargo proteins, the hydroxyl group of tyrosine forms essential hydrogen bonds with AP subunits, acting as a switch for vesicle recruitment. Substitution with alanine disrupts these interactions and impairs the trafficking pattern. In contrast, mutation of Y136 within the motif _136_YVDI_139_ had little impact on GP3 trafficking and viral replication, suggesting that this additional motif may be functionally redundant or only conditionally required. This premise is further supported by the greater sequence variability of the additional motif among PRRSV strains, particularly within PRRSV-1, indicating reduced evolutionary constraint and a lower likelihood that this motif functions as a primary sorting signal. It is also possible that the second motif modulates ER-to-Golgi trafficking efficiency, with its detectable effects becoming apparent only under conditions of increased virus particle production. From an evolutionary perspective, the functional asymmetry between the two YxxΦ motifs in PRRSV GP3 may reflect selective pressure to preserve one dominant trafficking signal while allowing flexibility in secondary motifs that adapt to changing secretory environments. The strong conservation of the motif _108_YAWL_111_ centered on Y108 across PRRSV isolates suggests an indispensable role in optimizing envelope protein routing, whereas the more variable additional motif may offer context-dependent modulation. Notably, substitutions at residues adjacent to the conserved tyrosines exerted pronounced effects on viral replication. Among the mutants, A109C and D138C exhibited delayed development of cytopathic effects and marked reductions in viral titers. Replacement with cysteine at these positions may disrupt secondary structure or hydrogen-bonding interactions, thereby compromising GP3 folding and reducing motif accessibility. Comparable sensitivity to disruption of YxxΦ-dependent processes has been observed in other viral systems. In HCV, for example, the YxxΦ motif within the core nucleocapsid protein is required for maintaining the structural integrity of non-enveloped capsid-like particles and for efficient clathrin-mediated endocytosis via AP-2 engagement, which subsequently directs particle trafficking to lipid droplets and enhances viral replication (Karamichali et al., 2017). Together, these findings suggest that even subtle sequence alterations affecting YxxΦ-associated structural or trafficking functions can lead to pronounced defects in downstream particle maturation and infectivity.

To date, no study is available addressing whether specific sequence motifs within PRRSV minor glycoproteins govern their intracellular targeting. Our work provides the first evidence that motif-dependent trafficking regulation operates in PRRSV minor envelope proteins and constrains viral fitness. Although YxxΦ-dependent GP3 trafficking is not essential for viral infectivity, it enhances the efficiency and fidelity of GP3 delivery to assembly sites, thereby optimizing the functional quality of progeny virions. More broadly, our findings highlight structural protein trafficking as an underappreciated determinant of PRRSV biology, uncovering a novel layer of control over intracellular localization and virion assembly.

## Data Availability

The raw data supporting the conclusions of this article will be made available by the authors, without undue reservation.

## Footnotes

1
https://www.ncbi.nlm.nih.gov/genbank/

2
http://elm.eu.org/

3
https://alphafoldserver.com/
